# First case report of spontaneous perinatal gastric perforation in premature neonate with potter sequence and syndrome

**DOI:** 10.1016/j.ijscr.2021.106297

**Published:** 2021-08-10

**Authors:** Katherine Lizeth Muñoz-Murillo, Willfrant Jhonnathan Muñoz-Murillo, Urías De Jesús Hernández-López, Laura María Aponte-Ceballos, Ivan David Lozada-Martínez, Sabrina Rahman

**Affiliations:** a School of Medicine, Universidad del Quindío, Carrera 15 #12N, Armenia, Colombia; b Department of Surgery, Universidad de Cartagena, Cra. 50 #24-120, Cartagena, Colombia; c Department of Medicine, Universidad de Cartagena, Cra. 50 #24-120, Cartagena, Colombia; d School of Medicine, Unidad Central del Valle del Cauca, Cra 27A ## 48-144, Tuluá, Colombia; e Medical and Surgical Research Center, School of Medicine, University of Cartagena, Cra. 50 #24-120, Cartagena, Colombia; f Department of Public Health, Independent University-Bangladesh, Dhaka, Bangladesh

**Keywords:** Potter sequence, Potter syndrome, Gastric perforation, Case report

## Abstract

**Introduction and importance:**

The Potter sequence is defined as a series of congenital defects related to severe oligohydramnios, associated with polycystic kidney disease, bilateral renal agenesis, pulmonary hypoplasia, obstructive uropathy and premature rupture of membrane, which compromises the life of the neonate sometime after birth. Within the evidence published so far, which is very little, no perforation of the gastrointestinal tract has been reported as a complication of this condition.

**Case presentation:**

Male neonate born preterm with prenatal diagnosis of pulmonary hypoplasia, polycystic renal dysplasia and severe oligohydramnios (Potter sequence), presented acute respiratory distress syndrome 10 min after birth, requiring mechanical ventilation and admission to the intensive care unit. During her stay in intensive care, he developed abdominal distension and presence of biliary content in the nasogastric tube. An abdominal X-ray was performed and showed signs of pneumoperitoneum, evidencing gastric perforation on exploratory laparotomy.

**Clinical discussion:**

Gastric perforation in neonates is a condition that causes high health costs, morbidity, high risk of mortality and disability, regardless of the cause. The management of gastric perforation in Potter syndrome, as well as any other complication, represents a challenge due to the prognosis of these patients. Renal failure and acute respiratory distress syndrome are disorders that compromise the function of various structures and organs such as the heart and brain.

**Conclusion:**

Gastric perforation is a possible complication of the Potter sequence or syndrome. In addition, there is no literature describing the benefits or disadvantages of specific surgical techniques in the resolution of perforation.

## Highlights

• Spontaneous perinatal gastric perforation is a complication of Potter's syndrome.
• Pathophysiological mechanism of gastric perforation in these cases is unknown.
• There is a lack of evidence on the management of potter's syndrome.

## 1 Introduction

The Potter sequence is defined as a series of congenital defects related to severe oligohydramnios, associated with polycystic kidney disease, bilateral renal agenesis, pulmonary hypoplasia, obstructive uropathy and premature rupture of membrane, which compromises the life of the neonate sometime after birth [1], [2], [3], [4], [5]. This condition generates changes in the physical appearance of the neonate, being characteristic the fascia composed by flattened nose, recessed chin, skin folds covering the corners of the eyes (epicanthal folds), and low-set abnormal ears [2].

So far, the exact cause behind this disorder is not known, however, the literature reports autosomal dominant, recessive and spontaneous forms [3]. The complications generated in this pathophysiological process are caused by the alteration of the amniotic fluid intake and urine excretion inside the uterus, generating a decrease in the volume of this fluid [1], [2]. Taking into account that the contribution of urine to the maintenance of this volume is more intense in the second and third trimester, it is in these times that the disorder is more prominent [1], [2]. Oligohydramnios generates compression of the fetus, limiting its movements and generating changes in physical appearance; and the increase in thoracic and intra-abdominal pressure does not allow adequate thoracic expiration, causing pulmonary hypoplasia [1], [2].

It occurs more frequently in males, has a general prevalence of 1 per 2000 to 5000 births and in primigravida fetuses [1], [4]. After birth, the terminology changes and becomes Potter syndrome [2], [6]. Renal failure, hydroelectrolyte imbalance, hydronephrosis, renal infection, respiratory distress syndrome, cardiac failure and gastrointestinal function disorder due to esophageal and intestinal agenesis are the most frequently reported complications of this syndrome [1], [2], [3], [4], [5], [6]. Within the evidence published so far, which is very little, no perforation of the gastrointestinal tract has been reported as a complication of this condition. The objective of this case is to report the first case of perinatal spontaneous gastric perforation as a complication in a patient with Potter sequence and syndrome. This case report followed the SCARE guidelines for its realization [7].

## 2 Presentation of case

16-year-old female patient, nulliparous and without relevant antecedents with ongoing pregnancy, who attended the emergency department of a maternal referral hospital in the city of Cartagena, Colombia; due to premature rupture of membranes, requiring vaginal labor induction for the presence of uterine contractions. The patient reported prenatal diagnosis of the fetus with pulmonary hypoplasia, polycystic renal dysplasia and severe oligohydramnios requiring amnioinfusion. She denied any history of smoking, alcohol, exposure to drugs, radiation or any other teratogenic factor. She denied any history of congenital disorders in her family.

**Fig. 1:**
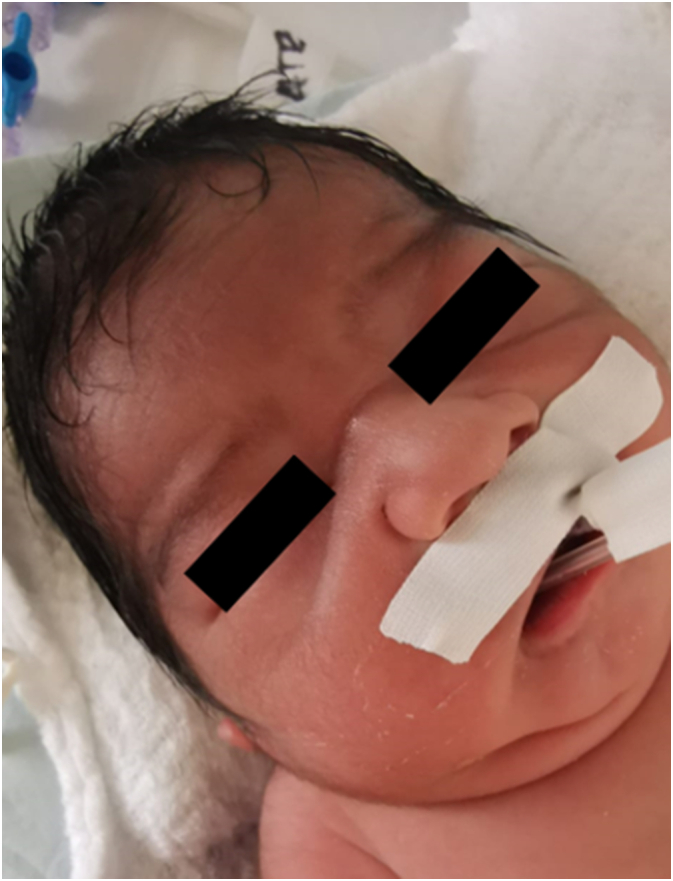
Photograph showing fascia characteristic of the Potter sequence and syndrome (flattened nose, recessed chin, skin folds covering the corners of the eyes and low-set abnormal ears).

**Fig. 2:**
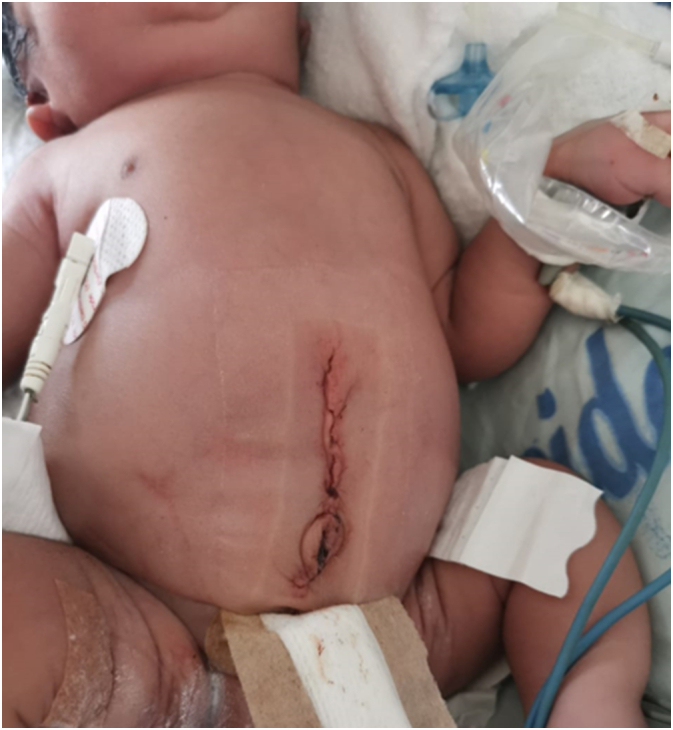
Postoperative photograph showing persistence of abdominal distention.

**Fig. 3:**
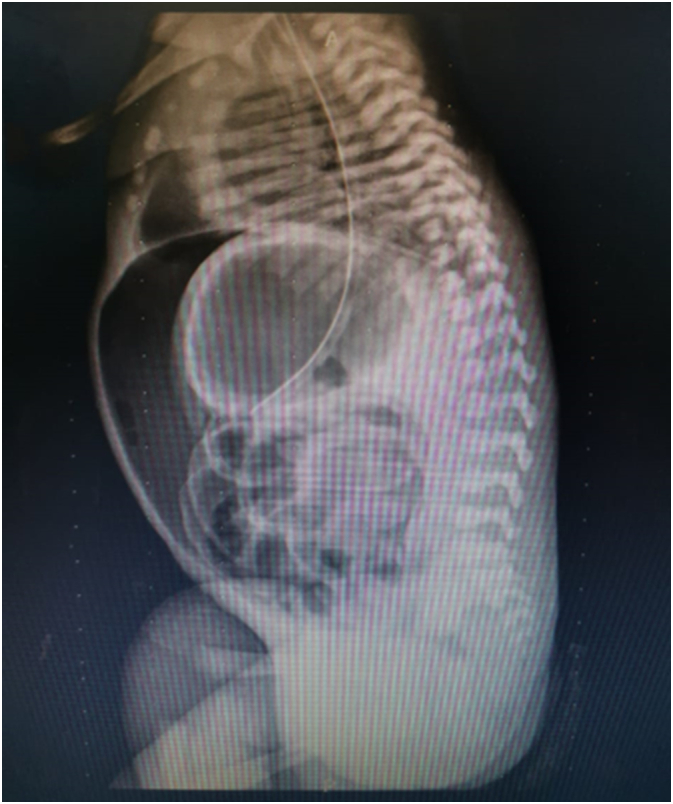
Abdominal X-ray showing signs of pneumoperitoneum.

We received a male neonate born preterm (32 weeks), with depressed nasal bridge and wide nose (Fig. 1), who presented acute respiratory distress syndrome 10 min after birth, requiring orotracheal intubation, mechanical ventilation and admission to the intensive care unit. During his stay in intensive care, he developed acute renal failure, presenting 2 days after birth with abdominal distention (Fig. 2) and presence of biliary content in the nasogastric tube. An abdominal x-ray was performed, showing signs of pneumoperitoneum (Fig. 3), so he was transferred to the pediatric surgery ward where an exploratory laparotomy was performed, showing a tear and wound in the posterior wall of the gastric greater curvature, compatible with perforation, and the lesion was corrected.

During the postoperative period he persisted with acute renal failure, hyperkalemia, metabolic acidosis, anasarca and anuria, dying seven days after birth.

## 3 Discussion

Gastric perforation in neonates is a condition that causes high health costs, morbidity, high risk of mortality and disability, regardless of the cause. Muscle defects or absence of the muscular layer of the gastric wall, iatrogenesis in the management of tracheoesophageal fistulas, hypoxia/ischemia, early sepsis, duodenal/jejunal obstruction, use of ibuprofen-paracetamol, esophageal atresia, administration of orogastric catheter, among others, are some of the causes reported in the literature [8], [9], [10], [11], [12], [13], [14], [15], [16]. Gastric perforation as a complication of Potter's syndrome has not been reported among the case series and case reports published so far [3], [4], [5], [17], [18], [19].

The management of gastric perforation in Potter syndrome, as well as any other complication, represents a challenge due to the prognosis of these patients. Renal failure and acute respiratory distress syndrome are disorders that compromise the function of various structures and organs such as the heart and brain. Curry et al. [17] performed in 1984 one of the most complete case series on Potter sequence, where they found that of 80 cases analyzed, 21.25% had bilateral renal agenesis, 47.5% had cystic dysplasia, and 25% had uropathy [17]. However, an important finding to highlight is that 15 of these patients had multiple congenital anomalies such as aneuploidy, autosomal recessive syndromes and causes that at the time were undetermined [17]. This is a point to consider about the prognosis and decision making in the intervention of complications of this syndrome, since it can be presumed that a large number of these cases present, in addition to clinical disorders, subclinical conditions that may not be compatible with the life of the neonate. However, it is an ethical dilemma to determine whether to perform interventions that increase the probability of survival of these patients, knowing the organ deficiencies they suffer from.

Fortunately, as science and technology advance, it is possible to opt for strategies that, although not applicable in all contexts, can change the prognosis. Miyahara et al. [19] reported the survival of a neonate with Potter sequence and low birth weight through long-term hemodialysis, peritoneal dialysis in case and renal transplantation [19]. This type of strategies with favorable results, is a demonstration of the need to design specialized centers to address rare diseases and genetic disorders, especially in low- and middle-income countries, where there are many difficulties for access to quality health services, mainly because pregnant women in rural and vulnerable areas, do not attend prenatal checkups, do not have adequate nutrition and are exposed to high-risk work for the fetus.

In the present case, gastrorrhaphy was performed with satisfactory results, however, the causes associated with Potter's syndrome caused death in the postoperative days. A fact that is necessary to analyze, especially when observing that the literature describes the placement of a nasogastric or orogastric tube as a cause of gastric perforation, is that in the present case the patient also had a nasogastric tube in place, however, the intraoperative finding was not compatible with a lesion related to the structure of the tube. As a limitation, histopathological study of the lesion is not performed due to institutional organizational changes due to the COVID-19 pandemic, and this service is used only in strictly necessary cases. Finally, the patient understood her baby's condition and was satisfied with the approach and effort made by the medical team. The present case reports for the first time gastric perforation as a complication of Potter's syndrome.

## 4 Conclusion

Gastric perforation is a possible complication of the Potter sequence or syndrome. The management of this complication is challenging due to the overall prognosis of these patients. In addition, there is no literature describing the benefits or disadvantages of specific surgical techniques in the resolution of perforation.

## Sources of funding

Non declared.

## Ethical approval

Hospital exempts ethics approval for reported cases.

## Consent written

Written informed consent was obtained from the patient for publication of this case report and accompanying images. A copy of the written consent is available for review by the Editor-in-Chief of this journal on request.

## Research registration

Not applicable.

## Guarantor

Sabrina Rahman. Department of Public Health, Independent University-Bangladesh, Dhaka, Bangladesh. sabrinaemz25@gmail.com.

## CRediT authorship contribution statement

All authors equally contributed to the analysis and writing of the manuscript.

## Declaration of competing interest

The authors declare that they have no known competing financial interests or personal relationships that could have appeared to influence the work reported in this paper.
